# Improving surgical team confidence for intraoperative cardiac arrest in non-standard positions (sitting beach chair and prone): a prospective in-situ simulation study with three-month follow-up

**DOI:** 10.1016/j.resplu.2026.101374

**Published:** 2026-06-02

**Authors:** Anna Sundelin, Andreas Wiklund, Mini Ruiz, Anders Stålman, Therese Djärv

**Affiliations:** a Capio Artro Clinic, Stockholm, Sweden; b Department of Physiology and Pharmacology, Section of Anaesthesia and Intensive Care, Karolinska Institutet, Stockholm, Sweden; c Department of CLINTEC, Karolinska Institutet, Stockholm, Sweden; d Department of MMK, Karolinska Institutet, Stockholm, Sweden; e Division for Clinical Medicine, Dept. of Medicine Solna, Karolinska Institutet, Stockholm, Sweden; f Internal Medicine & Emergency Medicine, Dept. Emergency Medicine. Karolinska University Hospital, Stockholm, Sweden

**Keywords:** In-situ simulation, CPR, Sitting beach chair position, Operating room, Surgical team, Interprofessional education, Patient safety, Role clarity, Critical event preparedness, Simulation curriculum design

## Abstract

**Background:**

Intraoperative cardiac arrest in non-standard positions, such as the sitting beach chair and prone positions, present unique challenges for surgical teams. Evidence on how to prepare teams for these rare events is limited. To evaluate whether a half-day, multi-station in situ simulation course was associated with changes in interprofessional surgical team members’ self-reported confidence and timed repositioning performance, and to describe self-reported application of course content to real patients three months afterwards.

**Methods:**

Prospective single-centre educational intervention at an elective orthopaedic unit. Interprofessional teams rotated through seven in situ stations during a 3.5-h course. Anonymous questionnaires on confidence in managing cardiac arrest were distributed before, immediately after and three months after the course. Exploratory transfer drills (sitting to supine and prone to supine) were timed within session. Confidence scores were analysed with the Mann-Whitney *U* test (primary comparison: Before versus three months). Effect sizes were reported as *r*, with |*r*| ≈ 0.1, 0.3 and 0.5 considered small, medium and large.

**Results:**

Sixty-three staff in five interprofessional teams participated. Questionnaire response rate was 74–92%, varied across domains. Self-reported confidence was higher at three months than before the course in all four domains (all *p* < 0.001), with medium to large effect sizes (*r* 0.39–0.68). The largest shift was for confidence in one’s own ability to manage cardiac arrest in the sitting beach-chair position (median 4 before versus 8 at three months). At 3 months, 48/56 (85.7%) respondents reported that course content had been of use for a real patient, including in situations other than cardiac arrest. Repositioning times showed a trend towards improvement in first-to-best transfer attempts, median ∼35 s (sitting to supine) and ∼30 s (prone to supine).

**Conclusion:**

A half-day, multi-station in situ simulation course at an elective surgical unit was associated with higher self-reported confidence at three months, and self-reported application of course content to real patients. Exploratory within-session transfer drills suggested improvements that are hypothesis-generating given the small number of teams. The design may be a feasible use of planned surgical downtime.

## Introduction

Cardiac arrest in the operating room (OR) is a rare but life-threatening event requiring rapid, coordinated team action.^1^ When patients are positioned in the sitting beach chair or prone positions, (common during orthopaedic and neurosurgical procedures), resuscitation efforts are often delayed due to complex repositioning, limited role clarity, and the presence of bulky surgical equipment such as microscopes or C-arm fluoroscopy units.^2^, ^3^, ^4^, ^5^ Current resuscitation guidelines, including the 2025 European Resuscitation Council recommendations for basic and advanced life support and for cardiac arrest in special circumstances, give little position-specific guidance for these scenarios.^6^, ^7^, ^8^ The International Liaison Committee on Resuscitation has not undertaken a systematic review of resuscitation in the sitting beach-chair position, although cardiac arrest in the prone position has been reviewed.^4^ Evidence on preparing surgical teams for such events through in-situ simulation is also limited and of very low certainty.^9^

In situ simulation has been shown to enhance team communication, identify latent safety threats, and improve resuscitation skills in hospital settings.^9^, ^10^, ^11^, ^12^, ^13^, ^14^, ^15^, ^16^, ^17^ However, few studies have explored its application in training surgical teams for CPR in non-supine patient positions, particularly the sitting beach chair setup, which presents unique ergonomic and logistical barriers.

This study aimed to evaluate whether in situ simulation was associated with surgical team members’ confidence in managing intraoperative cardiac arrest scenarios, and with repositioning times from sitting beach chair and prone positions to supine. We also examined the self-reported effects on real patients at three months and describe the development of local guidelines based on learnings from the intervention.

## Methods

### Design and setting

Prospective, single-centre educational intervention with repeated cross-sectional measurements at Capio Artro Clinic (Stockholm, Sweden), an elective surgery unit with seven operating rooms and 30 pre- and post-operative care beds. A single 3.5-h in-situ course was delivered once, on May 16, 2025, in the real operating rooms and perioperative areas. Interprofessional teams rotated through seven stations.

### Ethical considerations

An advisory opinion from the Swedish Ethical Review Authority (reference 2023-06315-01) concluded that the project had no ethical objections because only anonymous data were collected and participants were not subjected to any intervention beyond the course itself. Participation in the course was part of the unit’s ongoing staff education. Completion of the questionnaires was voluntary and anonymous; informed consent was indicated by questionnaire return.

### Participants

All staff employed at the unit was expected to attend the course as part of mandatory clinical training. Staff attending the course was eligible to complete the questionnaires. Fixed interprofessional teams consisted of participants from anesthesiology, orthopaedic surgery, operating room nursing and pre- and post-operative nursing.

### Intervention

**Fig. 1:**
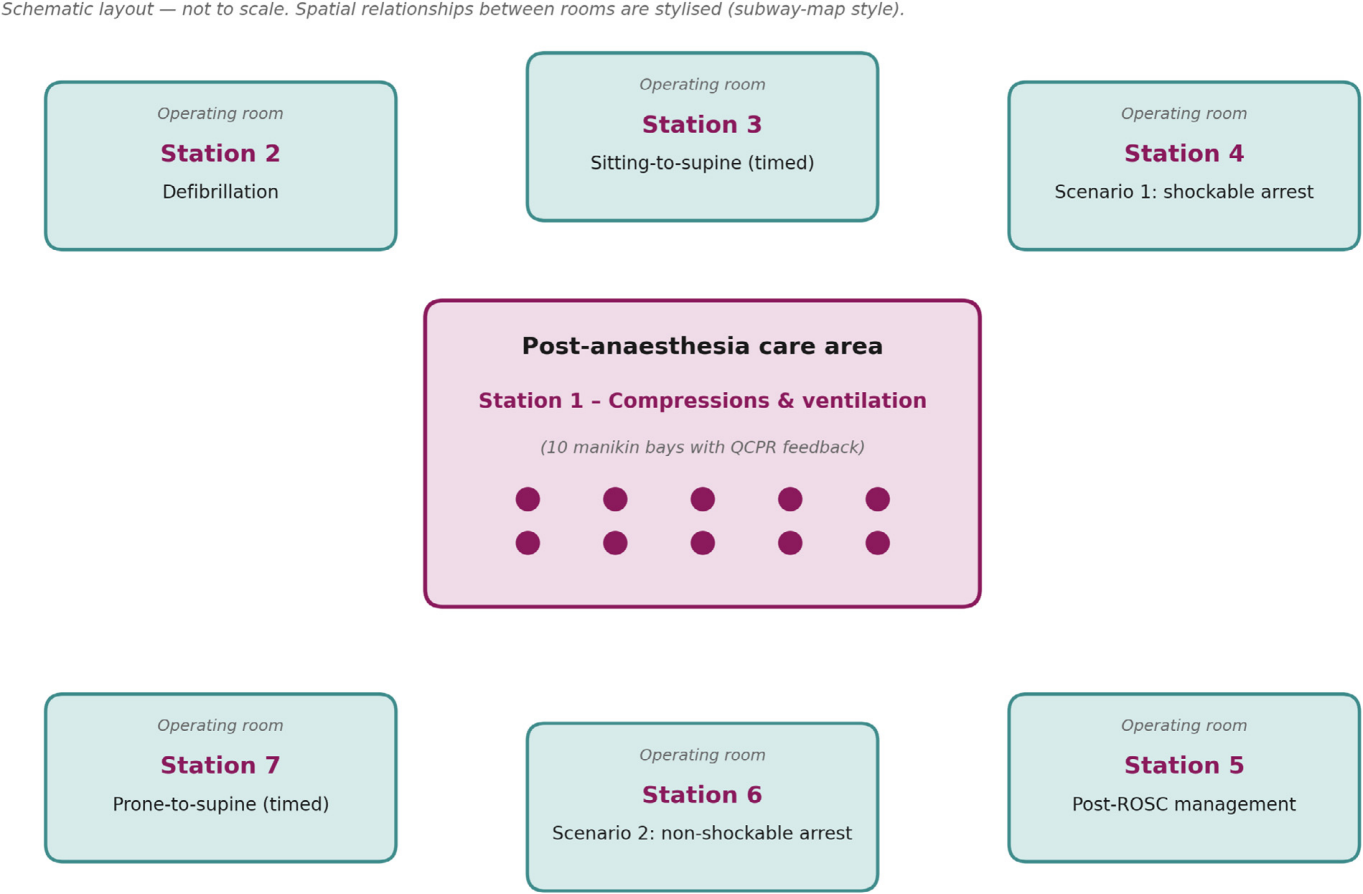
**Schematic layout of the seven simulation stations**. Station 1 (compressions and ventilation) was staged in the post-anaesthesia care area; Stations 2–7 ran concurrently in operating rooms.

**Fig. 2:**
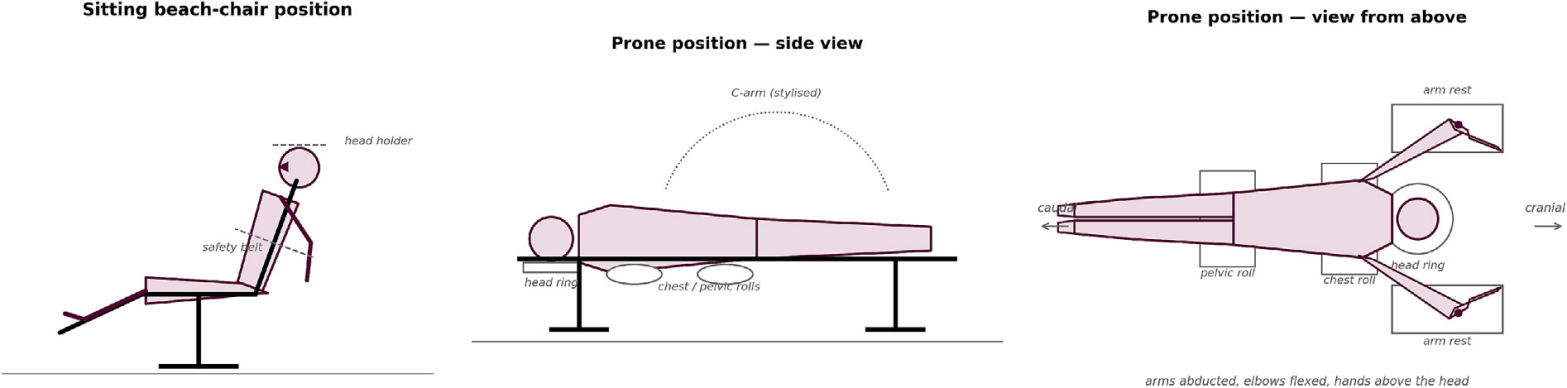
**Line-drawing schematic of the two non-standard surgical positions addressed by the course**. Left: sitting beach-chair position, with head holder and safety belt. Middle: prone position, side view, with head ring, chest and pelvic rolls and a stylised C-arm overhead (arms and arm boards omitted for clarity). Right: prone position, view from above, with the arms extended above the head as used at the unit.

The course was a half-day rotation of fixed interprofessional teams through the seven concurrent stations (approximately 25–30 min each), staffed by consultant anesthesiologists and senior operating room, anaesthesia and perioperative nurses. The schematic layout of the department during the course is shown in Fig. 1. The two non-standard surgical positions addressed are illustrated in Fig. 2.

Stations ran as follows:

1. **Compressions and ventilation.** Aim: deliberate individual practice of high-quality compressions and bag-mask ventilation with objective feedback. Modality: skill-trainer manikins with QCPR individual-feedback devices (Laerdal), staged in the post-anaesthesia care area. Staffing: two CPR instructors that are operating-room assistants. Measurement: QCPR individual feedback; summative assessment.
2. **Defibrillation.** Aim: ensure all participants could deliver a safe shock using the unit's defibrillators. Modality: pad placement and shock delivery using real in-situ clinical defibrillators; equipment and safety checks practised in the operating room (OR). Staffing: one anaesthesiologist and one anaesthesia nurse. Measurement: formative assessment.
3. **Sitting beach-chair to supine transfer (timed drill).** Aim: identify the fastest and safest team workflow for moving a secured sitting patient to the supine position ready for CPR. Modality: in-situ transfer of a healthy volunteer secured in the beach-chair position per unit routine (head and upper body fastened). Staffing: one anaesthesia nurse and one operating-room nurse. Measurement: elapsed time in seconds, first and best attempts.
4. **In-situ intraoperative cardiac arrest scenario 1 (shockable).** Aim: practise recognition and team management of a shockable arrest. Modality: simulated acute myocardial infarction with ventricular fibrillation, with emphasis on early detection, defibrillation, airway strategy, drug timing, team coordination, leadership, closed-loop communication, reversible causes, and return of spontaneous circulation (ROSC) detection and management. Staffing: one anaesthesiologist and one anaesthesia nurse. Measurement: formative assessment.
5. **Post-ROSC management.** Aim: discuss and rehearse the first critical minutes after ROSC. Modality: case-based micro-scenarios covering monitoring, haemodynamics, ventilation/oxygenation, antiarrhythmics, preventing re-arrest, documentation and handover. Staffing: one anaesthesiologist and one ICU/post-operative nurse. Measurement: formative assessment.
6. **In-situ intraoperative cardiac arrest scenario 2 (non-shockable, trauma CPR).** Aim: practise recognition and team management of a non-shockable, haemorrhage-driven arrest. Modality: simulated massive haemorrhage with pulseless electrical activity (PEA), with emphasis on early detection, airway management, haemorrhage control, activation of local transfusion and emergency protocols, reversible causes, team coordination, leadership, closed-loop communication, and ROSC detection and management. Staffing: two anaesthesiologists. Measurement: formative assessment.
7. **Prone to supine transfer (timed drill).** Aim: as for station 3, applied to the prone position with realistic equipment constraints. Modality: in-situ transfer of a healthy volunteer with a C-arm X-ray and a surgical microscope in place. Staffing: two anaesthesia nurses. Measurement: elapsed time in seconds, first and best attempts.

### Questionnaires and data collection

The questionnaire was adapted from an instrument used in a previous in situ simulation study from our group by developer consensus within the author group.^1^ In the current study, items about resuscitation in the sitting beach chair position were added. Items about prone position resuscitation were not added because the prone position literature has expanded substantially in recent years, whereas sitting position resuscitation remains less well-described. The instrument has not been formally validated.

Three questionnaires were administered: Before, Post (immediately after) and 3-month follow-up. The Before and Post questionnaires were paper-based and were distributed and collected by the station instructors immediately before and immediately after the course on the day of the intervention. The 3-month questionnaire was administered electronically by email three months after the course, with two reminders at one-week intervals. All questionnaires were anonymous and contained no identifier that would allow a given respondent’s answers to be linked across the three timepoints.

We asked participants to rate confidence in managing cardiac arrest, (defined broadly as recognition, team response, cardiopulmonary resuscitation (CPR), defibrillation, reversible causes, and ROSC management) in two clinical scenarios: general (i.e. in a standard supine position) and in the sitting beach-chair position. For brevity, domain labels use 'general CPR' and 'sitting (beach-chair) CPR' to indicate scenario context.

### Outcomes

#### Primary outcome (confidence)

Each respondent rated both confidence in their own ability to manage cardiac arrest and confidence in the team’s ability to manage cardiac arrest, both for general cardiac arrest and sitting cardiac arrest, on an 11-point scale (0–10; 10 = highest) at Before, Post, and 3 months. The prespecified primary comparison for each confidence domain was Before versus 3 months, reflecting the study’s focus on medium-term change.

#### Secondary outcomes

Before versus Post was a prespecified secondary comparison describing short-term change immediately after the course.

#### Subsequent use of skills

At three months, participants indicated whether they had applied any aspect of the training to a real patient (yes/no) and were invited to comment in free text.

#### Timed transfer performance (exploratory)

During the course, each team performed timed transfers sitting to supine and prone to supine. For each transfer type, we recorded the number of repetitions per team and the elapsed time (seconds) for the first and best attempts. These data were designated exploratory because of the small number of teams and observations.

### Analysis and statistics

Descriptive statistics were reported for each domain and timepoint as median (IQR) and range. Because questionnaires were anonymous and contained no linkable identifier, responses could not be paired across timepoints. The before, post and 3-month samples were therefore analysed as independent groups, using the two-sided Mann-Whitney *U* test. The primary comparison for each confidence domain was 3 months versus Before. The significance threshold was *α* = 0.05 and no multiplicity adjustment were applied given the four prespecified domains. Median differences between timepoints and 95% confidence intervals were estimated using the Hodges-Lehmann estimator with a rank-sum-based interval. Effect sizes were reported as *r* = |*Z*|/√(*n*_1_ + *n*_2_) with the sign indicating the direction of the change in medians; by convention, |*r*| ≈ 0.1, 0.3 and 0.5 are considered small, medium and large respectively.^18^

Self-reported real-patient application at 3 months was summarised as *n*/*N* (%) with a 95% Clopper-Pearson confidence interval. For transfer drills, per-team first- and best-attempt times were summarised descriptively (median, IQR, range) and were not tested given the small number of teams per drill.

Interpretation was based jointly on the direction and magnitude of the median shift (Hodges-Lehmann estimate with 95% CI), the effect size *r* and the *p*-value, rather than on statistical significance alone. On an 11-point confidence scale, a shift of one whole scale point in the group median was considered a clinically meaningful minimum.

Analyses were performed in Python, (Python Software Foundation 2023, Python Language Reference, version 3.11.2, libraries pandas, scipy, and matplotlib).

## Results

### Participants

Sixty-three staff attended the course (anesthesiology, orthopaedic surgery, OR and perioperative nursing). Response counts vary by domain/timepoint; 74–92% (tables in the Supplementary Appendix).

### Primary outcome: confidence

**Fig. 3:**
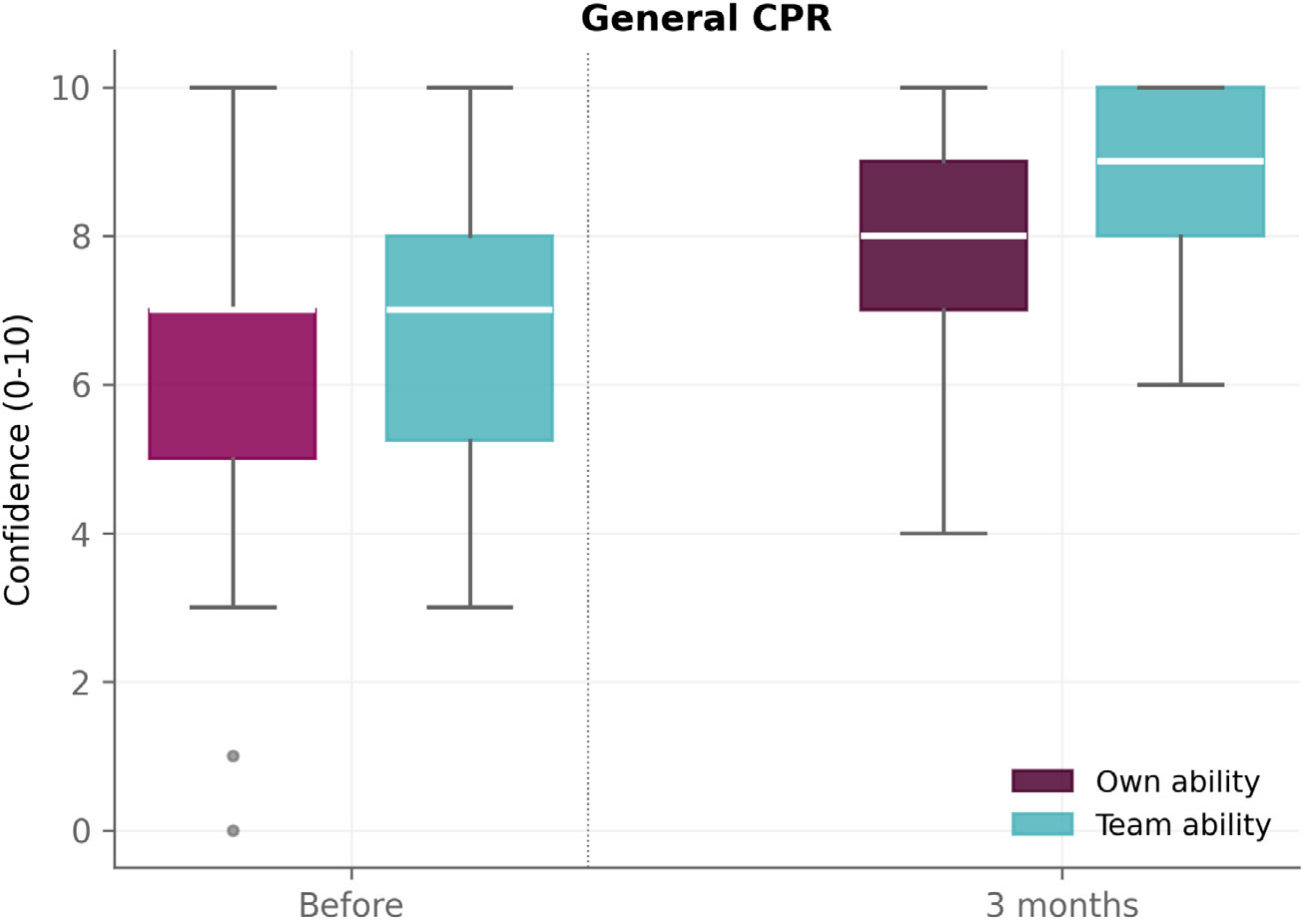
**Self-reported median confidence for general CPR, Before and at 3-month follow-up**. Scale: 0 = lowest, 10 = highest.

**Fig. 4:**
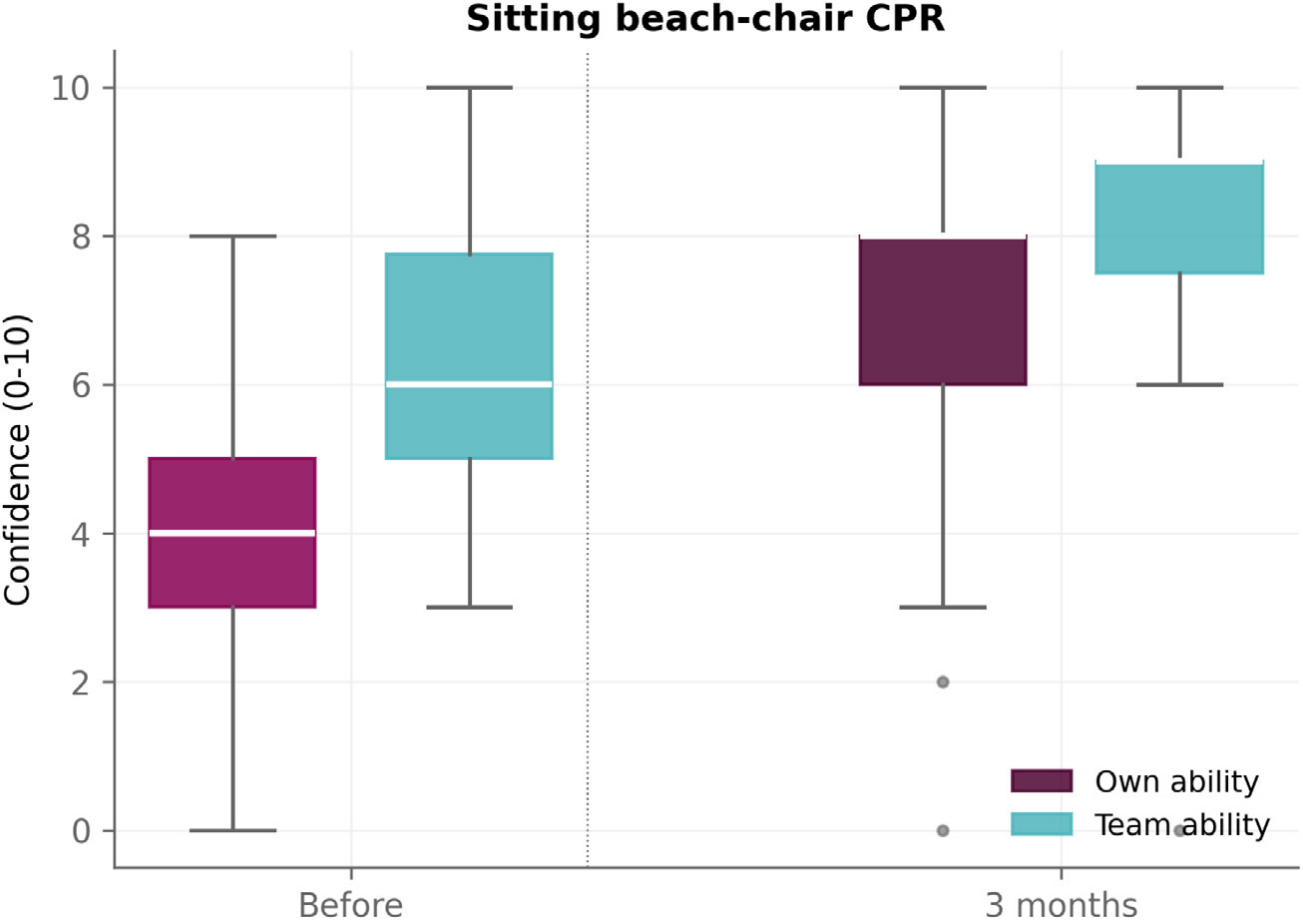
**Self-reported median confidence for CPR in the sitting beach-chair position, Before and at 3-month follow-up**. Scale as in Fig. 3.

Before to 3-month improvements were medium to large across all four domains (*r* = 0.4–0.7; all *p* < 0.001), (Table 1). Median (IQR [range]) confidence increased from 7 (5–7 [0–10]) to 8 (7–9 [4–10]) in one’s own ability to perform general CPR. Confidence in the team’s ability to perform CPR in general scenarios increased from 7 (5–8 [3–10]) to 9 (8–10 [6–10]). For sitting CPR confidence it improved from 4 (3–5 [0–8]) to 8 (6–8 [0–10]) in the participants’ own ability and from 6(5–8 [3–10]) to 9 (7.5–9 [0–10]) in the team’s ability. Boxplots of the primary comparison are shown I Fig. 3 (general CPR) and Fig. 4 (sitting beach chair CPR). Supplementary Fig. S1 shows Before versus Post.

**Table 1:** Self-reported median confidence by domain and timepoint.

**Domain**	**Timepoint**	***n***	**Median (IQR; range)**	**HL difference versus Before (95% CI)**	***p*-value**	***r***
Own ability, general CPR	Before	49	7 (5–7; 0–10)	–	–	–
Own ability, general CPR	Post	57	8 (8–9; 5–10)	2 (1.00–2.00)	<0.001	0.50
Own ability, general CPR	3 months	55	8 (7–9; 4–10)	1 (1.00–2.00)	<0.001	0.39

Team ability, general CPR	Before	50	7 (5–8; 3–10)	–	–	–
Team ability, general CPR	Post	57	9 (8–9; 7–10)	2 (1.00–2.00)	<0.001	0.53
Team ability, general CPR	3 months	55	9 (8–10; 6–10)	2 (1.00–2.00)	<0.001	0.50

Own ability, sitting CPR	Before	49	4 (3–5; 0–8)	–	–	–
Own ability, sitting CPR	Post	57	8 (7–8; 1–9)	4 (3.00–4.00)	<0.001	0.72
Own ability, sitting CPR	3 months	55	8 (6–8; 0–10)	3 (3.00–4.00)	<0.001	0.68

Team ability, sitting CPR	Before	46	6 (5–8; 3–10)	–	–	–
Team ability, sitting CPR	Post	57	9 (8–9; 6–10)	2 (2.00–3.00)	<0.001	0.63
Team ability, sitting CPR	3 months	55	9 (8–9; 0–10)	2 (1.00–3.00)	<0.001	0.53

Each participant rated confidence in their own ability and in the team's ability to manage cardiac arrest, for general CPR and for CPR in the sitting beach-chair position, on an 11-point numerical scale (0 = lowest, 10 = highest). Prespecified primary comparison: Before versus 3 months, Mann-Whitney *U* test, two-sided, *α* = 0.05. Post versus Before is the prespecified secondary comparison. Hodges-Lehmann estimator for median differences with 95% CI. Effect size *r* = |*Z*|/√(*n*_1_ + *n*_2_), signed to match direction of median change; by convention |*r*| ≈ 0.1, 0.3 and 0.5 are small, medium and large. IQR = interquartile range.

### Real-patient application at 3 months

Respondents reported in 48/56 (85.7%; 95% CI 73.8–93.6) follow-up questionnaires that course learning had been beneficial in clinical care within three months. In the comments, clearer role allocation, improved team efficiency, faster positioning changes, and enhanced communication were mentioned. Among participants who did not report real-patient benefit at three months, several noted that no relevant emergency had occurred to allow practice of the course content.

### Exploratory outcome: transfer times

**Fig. 5:**
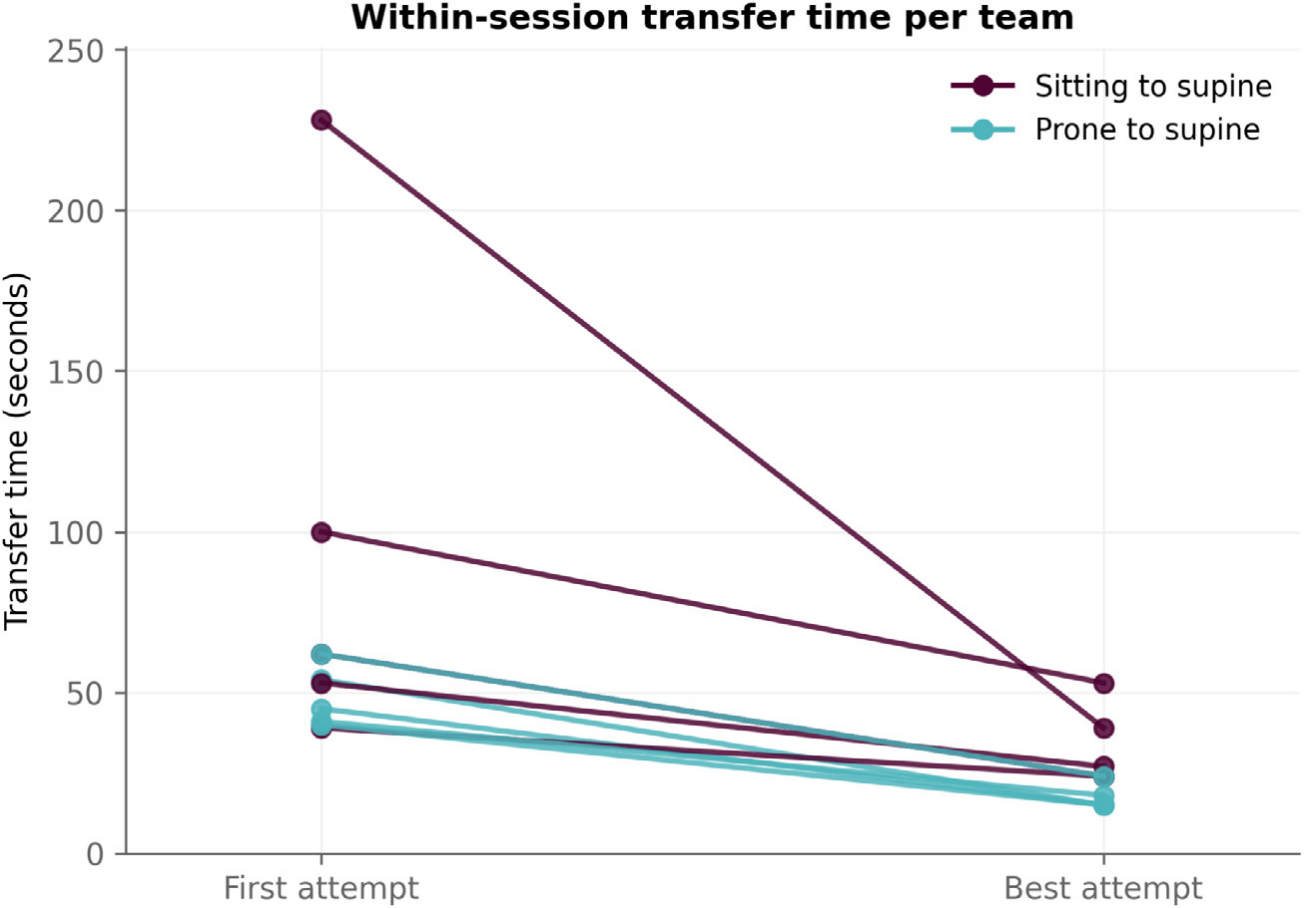
**Slope chart showing per-team elapsed time (s) from first to best attempt for sitting-to-supine (left) and prone-to-supine (right) transfer drills. Each line represents one team (*n* = 5 per drill). Number of attempts per team: 2–3 for sitting-to-supine; 3–5 for prone-to-supine. Drills were exploratory and no inferential testing was performed**.

Within-session sitting to supine and prone to supine timings showed sizeable median first to best reductions (∼40 s and ∼30 s, respectively) when comparing each team’s first to best attempt. Sitting to supine performed 2–3 attempts per team. Prone to supine performed 3–5 attempts per team. Given the small number of teams (*n* = 5 per drill), these data were designated exploratory and no inferential testing was performed. A descriptive slope-chart is shown in Fig. 5.

### Local implementation

Following the course, and drawing on observations from the in-situ simulations, the unit developed formal, written guidelines for repositioning from sitting and prone to supine, including role assignments and interaction with surgical equipment, (e.g. operating table, C-arm and microscope). A local guideline on managing intraoperative cardiac arrest was also prepared. The guidelines are provided as Supplementary Material. This was not a prespecified outcome and is reported descriptively.

## Discussion

This study suggests that a half-day, multi-station in situ simulation course at an elective orthopaedic surgery unit is associated with higher self-reported confidence at three months compared with before the course, across both general cardiac arrest and cardiac arrest in the sitting beach chair position. Most respondents at three months reported that something they had learned had been of use for a real patient, including in situations other than cardiac arrest. Within session transfer drills were exploratory and hypothesis-generating.

The sitting beach chair scenario is rarely addressed in resuscitation training literature. Confidence levels remained elevated at 3-month follow-up, and most respondents at that timepoint reported having applied something from the course to a real patient, including outside cardiac arrest. This is consistent with prior studies of in situ simulation in hospital settings showing simulation’s effect on latent safety threat identification and non-technical skill development.^1^, ^10^, ^11^, ^13^, ^14^, ^16^, ^17^, ^19^, ^20^

### Strengths and limitations

Strengths are the shown real-world impact, both on a systems level and real patient benefit. The project led to the creation of formal local guidelines, a tangible systems-level outcome also described in previous studies.^1^, ^9^, ^14^ These protocols provided clear role assignments and stepwise repositioning plans, including strategies to manage complex equipment like the C-arm and surgical microscope, and addressed equipment-specific challenges at our unit.

The design of the intervention (seven rotating simulation stations across a half-day) offered high engagement and realism. This structured multi-station, interdisciplinary format is novel in the context of surgical team CPR training and may serve as a model for scalable implementation in other high-risk environments.

Limitations include the small sample size and the single-centre nature of the study. Retention of resuscitation skills and confidence typically decays over months, and our study did not standardise or track interim refreshers between baseline and the 3-month-followup. Confidence is a useful proximal outcome, but it is not synonymous with competence. Self-ratings can over- or underestimate actual performance. The questionnaire is not formally validated. The described differences in confidence are between distributions and not within-person changes. The inability to link responses across timepoints is an important limitation. Because the questionnaires were anonymous and carried no identifier, the Before, Post and three-month samples comprise overlapping but not identical sets of respondents, and different individuals may have answered at each timepoint. Response was also incomplete and varied across timepoints and domains (74–92%). Any observed change could therefore be confounded by differential response and non-response, and we could neither estimate within-person change nor adjust for incomplete follow-up.

### Future research

Future research should explore objective measures of clinical performance and patient outcomes. Examples of this could be time-to-first shock in sitting or prone operation positions. Another line of potential studies could be pairing self-reported confidence with observational competence measures such as timing of repositioning or CPR manikins with live feedback metrics. Applicability to other surgical specialties and operation positions would also be of interest investigating.

## Conclusion

A half-day, multi-station in situ simulation course at an elective surgical unit was associated with higher self-reported confidence at three months and self-reported application of course content to real patients. Exploratory within-session transfer drills suggested improvements that are hypothesis-generating given the small number of teams. The design may be a feasible use of planned surgical downtime.

## Editorial board declaration

Therese Djärv is a member of the editorial board of Resuscitation Plus. She had no role in the handling of this manuscript.

## CRediT authorship contribution statement

**Anna Sundelin:** Writing – review & editing, Writing – original draft, Visualization, Validation, Resources, Project administration, Methodology, Investigation, Funding acquisition, Formal analysis, Data curation, Conceptualization. **Andreas Wiklund:** Writing – review & editing, Methodology, Conceptualization. **Mini Ruiz:** Writing – review & editing, Methodology, Conceptualization. **Anders Stålman:** Writing – review & editing, Supervision, Resources. **Therese Djärv:** Writing – review & editing, Supervision, Conceptualization.

## Funding

Departmental education time; no external funding.

## Declaration of competing interest

None declared.

## Data Availability

De-identified data and analysis code available on reasonable request.
