# Supplementary material for: Improving surgical team confidence for intraoperative cardiac arrest in non-standard positions (sitting beach chair and prone): a prospective in-situ simulation study with three-month follow-up
Source: Resusc Plus. 2026 Jun 2;30:101374. doi: 10.1016/j.resplu.2026.101374 (PMC13279733; doi:10.1016/j.resplu.2026.101374)
Supplement: Supplementary Data 1 [file mmc1.docx]

# Improving Surgical Team Confidence for Intraoperative Cardiac Arrest in Non-Standard Positions (Sitting Beach Chair and Prone): A Prospective In-Situ Simulation Study with Three-Month Follow-Up

## Supplementary Appendix

Table of Contents

[Improving Surgical Team Confidence for Intraoperative Cardiac Arrest in Non-Standard Positions (Sitting Beach Chair and Prone): A Prospective In-Situ Simulation Study with Three-Month Follow-Up 1](#_Toc231802723)

[Supplementary Appendix 1](#_Toc231802724)

[Figure S1. *Self-reported median confidence before and immediately after the course (Before vs Post) for general CPR and for CPR in the sitting beach-chair position.* 1](#_Toc231802725)

[Table S1. Descriptive statistics and between-timepoint comparisons of self-reported median confidence. 2](#_Toc231802726)

[Local guidelines developed after the course 3](#_Toc231802727)

[In case of cardiac arrest in beach-chair position (sitting patient) 3](#_Toc231802728)

[In case of cardiac arrest in prone position 5](#_Toc231802729)

[In case of cardiac arrest during ongoing anaesthesia 7](#_Toc231802730)

## Figure S1. *Self-reported median confidence before and immediately after the course (Before vs Post) for general CPR and for CPR in the sitting beach-chair position.*


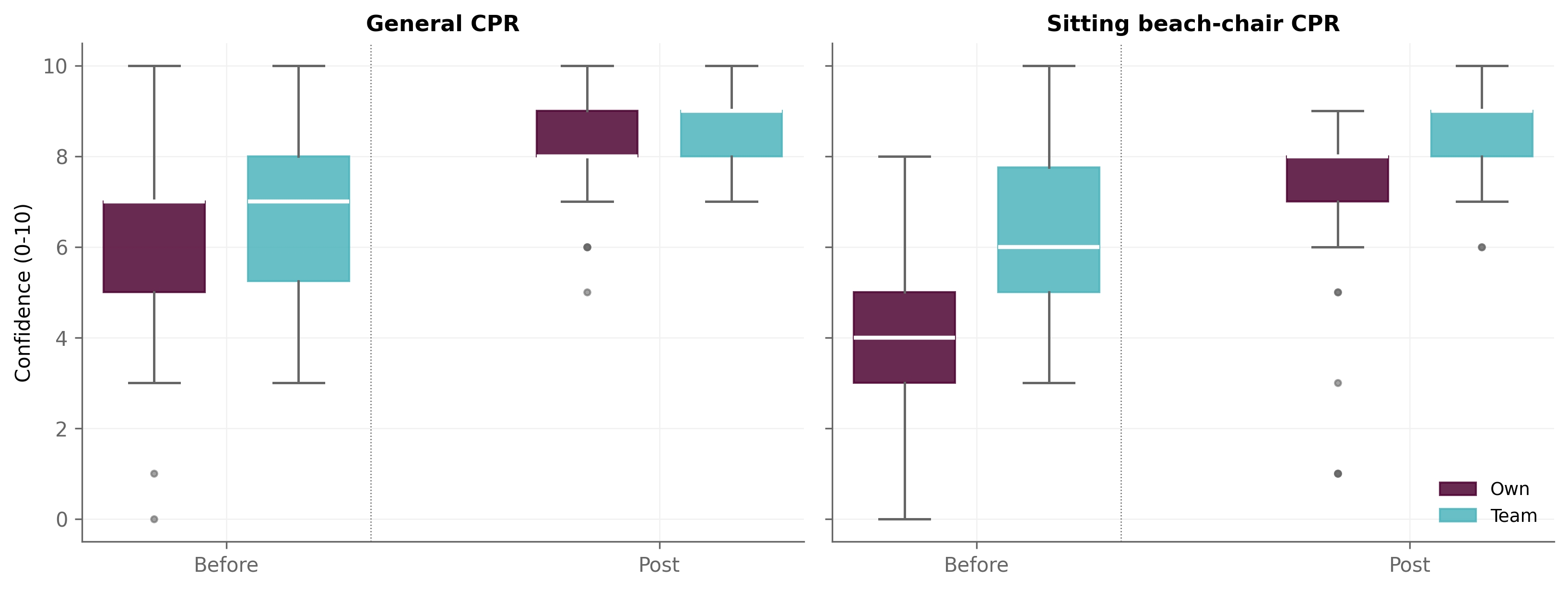


## Table S1. Descriptive statistics and between-timepoint comparisons of self-reported median confidence.

*Self-reported confidence for general CPR and for CPR in the sitting beach-chair position, for own ability and team ability, on an 11-point scale (0 = lowest, 10 = highest). Because questionnaires were anonymous and could not be linked across timepoints, Before, Post and 3-month samples were analysed as independent groups using the two-sided Mann-Whitney U test. Median differences are Hodges-Lehmann estimates with rank-sum-based 95% confidence intervals; effect size r = |Z| / √(n_1_ + n_2_), signed for direction; |r| benchmarks ≈ 0.1 small, 0.3 medium, 0.5 large.*

| Domain | Timepoint | n | Mean | SD | Median | IQR | Range | HL diff vs Before (95% CI) | Z | p-value | r (signed) |
| --- | --- | --- | --- | --- | --- | --- | --- | --- | --- | --- | --- |
| Own general CPR | Before | 49 | 6.39 | 2.02 | 7.0 | 5.0–7.0 | 0–10 | – | – | – | – |
| Own general CPR | Post | 57 | 8.11 | 0.99 | 8.0 | 8.0–9.0 | 5–10 | 2.0 (1.00 to 2.00) | 5.19 | <0.001 | 0.50 |
| Own general CPR | 3 months | 55 | 7.84 | 1.40 | 8.0 | 7.0–9.0 | 4–10 | 1.0 (1.00 to 2.00) | 4.02 | <0.001 | 0.39 |
| Team general CPR | Before | 50 | 7.02 | 1.80 | 7.0 | 5.2–8.0 | 3–10 | – | – | – | – |
| Team general CPR | Post | 57 | 8.82 | 0.83 | 9.0 | 8.0–9.0 | 7–10 | 2.0 (1.00 to 2.00) | 5.52 | <0.001 | 0.53 |
| Team general CPR | 3 months | 55 | 8.78 | 1.18 | 9.0 | 8.0–10.0 | 6–10 | 2.0 (1.00 to 2.00) | 5.12 | <0.001 | 0.50 |
| Own sitting CPR | Before | 49 | 3.86 | 1.93 | 4.0 | 3.0–5.0 | 0–8 | – | – | – | – |
| Own sitting CPR | Post | 57 | 7.35 | 1.89 | 8.0 | 7.0–8.0 | 1–9 | 4.0 (3.00 to 4.00) | 7.38 | <0.001 | 0.72 |
| Own sitting CPR | 3 months | 55 | 7.13 | 1.95 | 8.0 | 6.0–8.0 | 0–10 | 3.0 (3.00 to 4.00) | 6.92 | <0.001 | 0.68 |
| Team sitting CPR | Before | 46 | 6.37 | 1.58 | 6.0 | 5.0–7.8 | 3–10 | – | – | – | – |
| Team sitting CPR | Post | 57 | 8.49 | 1.02 | 9.0 | 8.0–9.0 | 6–10 | 2.0 (2.00 to 3.00) | 6.37 | <0.001 | 0.63 |
| Team sitting CPR | 3 months | 55 | 8.16 | 1.71 | 9.0 | 7.5–9.0 | 0–10 | 2.0 (1.00 to 3.00) | 5.36 | <0.001 | 0.53 |

## Local guidelines developed after the course

*English translations of the local quick-reference checklists used at the unit. Structure and wording follow the original Swedish documents. Pages listing cardiac arrest in the beach-chair position, prone position and during ongoing anaesthesia are intended to be printed and made available in the relevant theatres.*

### In case of cardiac arrest in beach-chair position (sitting patient)

**Circulating staff (in the room)**

- Activate the emergency alarm.
- Ask: "Who is doing compressions?"
- Release the head straps.
- Release the safety belt.
- Take the table control.
- Lower the backrest as soon as the head is freed.
- Slide the patient down.
- Optimise ergonomics (lower the table? fetch a step stool?).

**Anaesthesia nurse**

- "Cardiac arrest – call for help."
- 100% oxygen; pause anaesthesia.
- Hold the head; stabilise the neck.
- Initiate sliding the patient downward so the head rests on the table.
- "Does everyone have a grip? 1–2–3 pull."
- Hand the head over to other anaesthesia staff.
- Give a brief handover.

**Scrub nurse**

- Undrape.
- Provide compressions or relieve the person doing compressions.
- Raise the shoulder support.
- Slide the patient downward.
- Cover the wound.

**Surgeon**

- Undrape.
- Provide compressions or relieve the person doing compressions.
- Slide the patient downward.
- Cover the wound.

**Responding team — anaesthesia personnel**

- Relieve the person holding the head to enable a brief handover.
- Airway lead: 30:2 or continuous compressions until otherwise ordered by the anaesthesiologist.
- Pulse check (carotid) when an organised rhythm appears.
- EtCO₂ monitoring.
- Ventilation: low airway pressures; respiratory rate 10/min until otherwise ordered by the anaesthesiologist.

**Responding team — other personnel**

- Relieve chest compressions.
- Call 112 (record the time).
- Apply defibrillation pads.
- Start rhythm analysis as soon as pads are attached.
- Assign roles: "Who is ____?" — defibrillation lead, recorder, medication lead, IV/IO access lead, leader with overview and reversible causes.

**Anaesthesiologist**

- Avoid hands-on tasks.
- Maintain helicopter view (overall coordination).
- Delegate to the CPR-algorithm team leader if needed.
- Reassess ABCDE.
- Treat reversible causes.

**Notes – beach position, sitting patient**

- When positioning for surgery: fasten the safety belt with the buckle at the back for easy release.
- It is essential to free the patient's head from the helmet before flattening the table to avoid neck injury.
- Move the patient down toward the foot end as soon as possible so the head is supported and CPR can continue in the supine position.
- This protocol is written for cardiac arrest but also applies to other acute situations where the patient must quickly be brought to the supine position.

### In case of cardiac arrest in prone position

**Circulating staff (in the room)**

- Activate the emergency alarm.
- Ask: "Who is doing compressions?"
- Move the microscope / C-arm away.
- Remove the arm board.
- Turn the patient.
- If alone: relieve compressions immediately after turning.
- Optimise ergonomics (lower the table? fetch a step stool?).

**Anaesthesia nurse**

- "Cardiac arrest – call for help."
- 100% oxygen; pause anaesthesia.
- Hold the head.
- Initiate the turn.
- "Does everyone have a grip? 1–2–3 turn."
- Hand the head over to other anaesthesia staff.
- Give a brief handover.

**Scrub nurse**

- Cover the surgical wound.
- Provide compressions or relieve the person doing compressions.
- Make space for the turning board.
- If Mayfield clamp in use: release the head from the table (surgeon holds the head).
- Turn the patient.

**Surgeon**

- Ensure the turn is safe (neck stabilised).
- Cover the surgical wound.
- Provide compressions or relieve the person doing compressions.
- If Mayfield clamp in use: take control of the head.
- Make space for the turning board.
- Turn the patient.

**Responding team — anaesthesia personnel**

- Relieve the person holding the head to enable a brief handover.
- Airway lead: 30:2 or continuous compressions until otherwise ordered by the anaesthesiologist.
- Keep track of tubes and cables.
- Pulse check (carotid) when an organised rhythm appears.
- EtCO₂ monitoring.
- Ventilation: low airway pressures; respiratory rate 10/min until otherwise ordered by the anaesthesiologist.

**Responding team — other personnel**

- Fetch the turning board.
- Place the turning board nearest the doors.
- Turn the patient.
- Relieve chest compressions.
- Call 112 (record the time).
- Apply defibrillation pads.
- Start rhythm analysis as soon as pads are attached.
- Assign roles: defibrillation lead, recorder, medication lead, IV/IO access lead, leader with overview and reversible causes.

**Anaesthesiologist**

- Avoid hands-on tasks.
- Maintain helicopter view.
- Delegate to the CPR-algorithm team leader if needed.
- Reassess ABCDE.
- Treat reversible causes.

**Notes – prone position**

- The circulating team moves the microscope / C-arm to the far corner toward the room exit.
- The turning board must always be kept in the preparation room, unlocked.
- If compressions are ongoing on the side nearest the doors (where the turning board must go), switch compressions to the other side promptly so the board can be placed.
- With a Mayfield head clamp: do not compress the chest while the head is fixed to the table due to risk of neck injury. Release the head quickly: leave the Mayfield attached to the patient but detach it from the table.
- For cervical or high thoracic surgery (around T4 or above) where spinal cord injury would be incompatible with life, the surgeon must state clearly whether turning, defibrillation and compressions are safe.

### In case of cardiac arrest during ongoing anaesthesia

**Circulating staff (in the room)**

- Activate the emergency alarm.
- Ask: "Who is doing compressions?"
- Optimise ergonomics (lower the table? fetch a step stool? remove arm boards?).

**Anaesthesia nurse**

- "Cardiac arrest – call for help."
- 100% oxygen; pause anaesthesia.
- Hand over holding the head to enable a brief handover.

**Scrub nurse**

- Order depends on the patient and situation.
- Undrape.
- Provide compressions or relieve the person doing compressions.
- Cover the wound.

**Surgeon**

- Order depends on the patient and situation.
- Undrape.
- Provide compressions or relieve the person doing compressions.
- Cover the wound.

**Responding team — anaesthesia personnel**

- Relieve the person holding the head to enable a brief handover.
- Airway lead: 30:2 or continuous compressions.
- Pulse check (carotid) when an organised rhythm appears.
- EtCO₂ monitoring.
- Ventilation: low airway pressures; respiratory rate 10/min.

**Responding team — other personnel**

- Relieve chest compressions.
- Call 112 (record the time).
- Apply defibrillation pads.
- Start rhythm analysis as soon as pads are attached.
- Assign roles: defibrillation lead, recorder, medication lead, IV/IO access lead, helicopter view (overview and reversible causes).

**Anaesthesiologist**

- Avoid hands-on tasks.
- Maintain helicopter view.
- Delegate to the CPR-algorithm team leader if needed.
- Reassess ABCDE.
- Treat reversible causes.
